# Renal capsule metastasis from renal pelvic cancer: a case report

**DOI:** 10.1186/s12894-018-0324-9

**Published:** 2018-03-01

**Authors:** Yasuyuki Kobayashi, Hiroki Arai, Masahito Honda, Takashi Matsumoto, Kyotaro Yoshida

**Affiliations:** 1 Departments of Urology, Kinki Central Hospital of Mutual Aid Association of Public Teachers, 3-1 Kurumazuka, Itami, Hyogo 664-8533 Japan; 2 Surgery, Kinki Central Hospital of Mutual Aid Association of Public Teachers, 3-1 Kurumazuka, Itami, Hyogo 664-8533 Japan; 3 Pathology, Kinki Central Hospital of Mutual Aid Association of Public Teachers, 3-1 Kurumazuka, Itami, Hyogo 664-8533 Japan

**Keywords:** Breast cancer, Renal capsule metastasis, Renal cell cancer, Renal pelvic cancer, Urothelial carcinoma

## Abstract

**Background:**

Metastatic renal cancers are relatively common. Most are metastases to the renal parenchyma via a hematogenous route and are derived from lung, breast, and gastrointestinal cancer, malignant melanoma, and hematologic malignant cancer. However, little is known about renal capsule metastasis from other cancers.

**Case presentation:**

We report a 71-year-old woman with breast cancer who was treated with endocrine therapy. She presented with gross hematuria and was diagnosed as having right renal pelvic cancer and renal cell cancer. She underwent right laparoscopic radical nephroureterectomy. Pathological findings revealed right pelvic cancer and renal capsule metastasis.

**Conclusion:**

Renal capsule metastasis derived from renal pelvic cancer is very rare. When diagnosing renal capsule cancer, we believe that renal capsule metastasis should also be taken into consideration. Clinical and radiological differential diagnosis of renal capsule metastasis from renal cell cancer and primary renal capsule cancer is difficult. Assessment of the histopathological findings of the surgical specimens seems to be the only realistic approach to achieving the correct diagnosis.

## Background

Metastatic renal cancer is relatively common and is mainly derived from lung, breast, and gastrointestinal cancer, malignant melanoma, and hematologic malignant cancer [1–4]. Almost all of the metastases are to the renal parenchyma, and to our best knowledge, there is no case report describing metastasis to the renal capsule. We report a rare case of renal capsule metastasis derived from renal pelvic cancer.

## Case presentation

A 71-year-old woman was diagnosed as having breast cancer (left breast, invasive lobular carcinoma, T4cN3cM1, Stage IV) in September 2014 and was treated with endocrine therapy (exemestane 25 mg/day). Gross hematuria was pointed out in January 2015, and hematuria was detected by urinalysis. Her past history included hypertension and diabetes mellitus but no history of smoking. Urinary cytology was Class III (Papanicolaou classification) [5]. Blood tests showed a hemoglobin of 12.0 g/dL, serum creatinine of 0.87 mg/dL, lactate dehydrogenase of 251 U/L, aspartate aminotransferase of 18 U/L, and alanine aminotransferase of 6 U/L. Computed tomography (CT) showed a hypovascular mass 25 mm diameter in the right renal pelvis and a hypervascular mass of 22 mm in diameter in the upper pole of the right kidney. The hypervascular mass showed a contrast effect in the early phase. There were no signs of metastasis in the lung, liver, or abdominal lymph nodes. A retrograde pyelogram showed a filling defect in the right renal pelvis, and catheterized urine cytology was class III (Papanicolaou classification). No obvious findings were observed on radiographic imaging of the ureter.

We diagnosed her as having right renal pelvic cancer (cT3N0M0) and right renal cell cancer (cT1aN0M0). Because her metastatic breast cancer prognosis was expected to be relatively good, she thus underwent right laparoscopic radical nephroureterectomy via a retroperitoneal approach. As described below, we diagnosed right pelvic cancer and renal capsule metastasis.

**Fig. 1:**
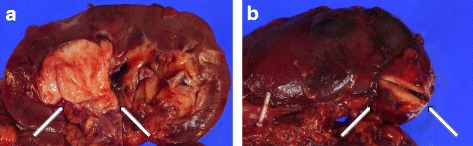
Gross appearance of the right kidney. **a** Renal pelvic cancer occupying the renal pelvis and invading the renal parenchyma (arrows). **b** The renal capsule cancer coating the renal capsule was easily peeled off from the renal parenchyma (arrows)

Macroscopically, the renal pelvic tumor was 4 cm in diameter, yellowish white, and soft. It was located in the right renal pelvis and infiltrated into the parenchyma. The other renal tumor was 2.5 cm in diameter, well-circumscribed, yellowish white, and hard (Fig. 1). It was coated with renal capsular tissue and was easily decapsulated from the renal parenchyma. This tumor was considered to be a renal capsule tumor. The ureteral wall was thickened, but its mucosa was normal.

Microscopically, the renal pelvic tumor invaded the parenchyma but not the capsule beyond the parenchyma. The renal capsule tumor was circumscribed by the renal capsule and did not invade neighboring tissue. Both tumor cells were similar and had eosinophilic cytoplasm, round nuclei, and an alveolar pattern of growth, and they were concordant with urothelial carcinoma. Tumor cells of the ureter had eosinophilic cytoplasm, round nuclei, and a trabecular pattern of growth similar to those of the breast cancer cells.

**Fig. 2:**
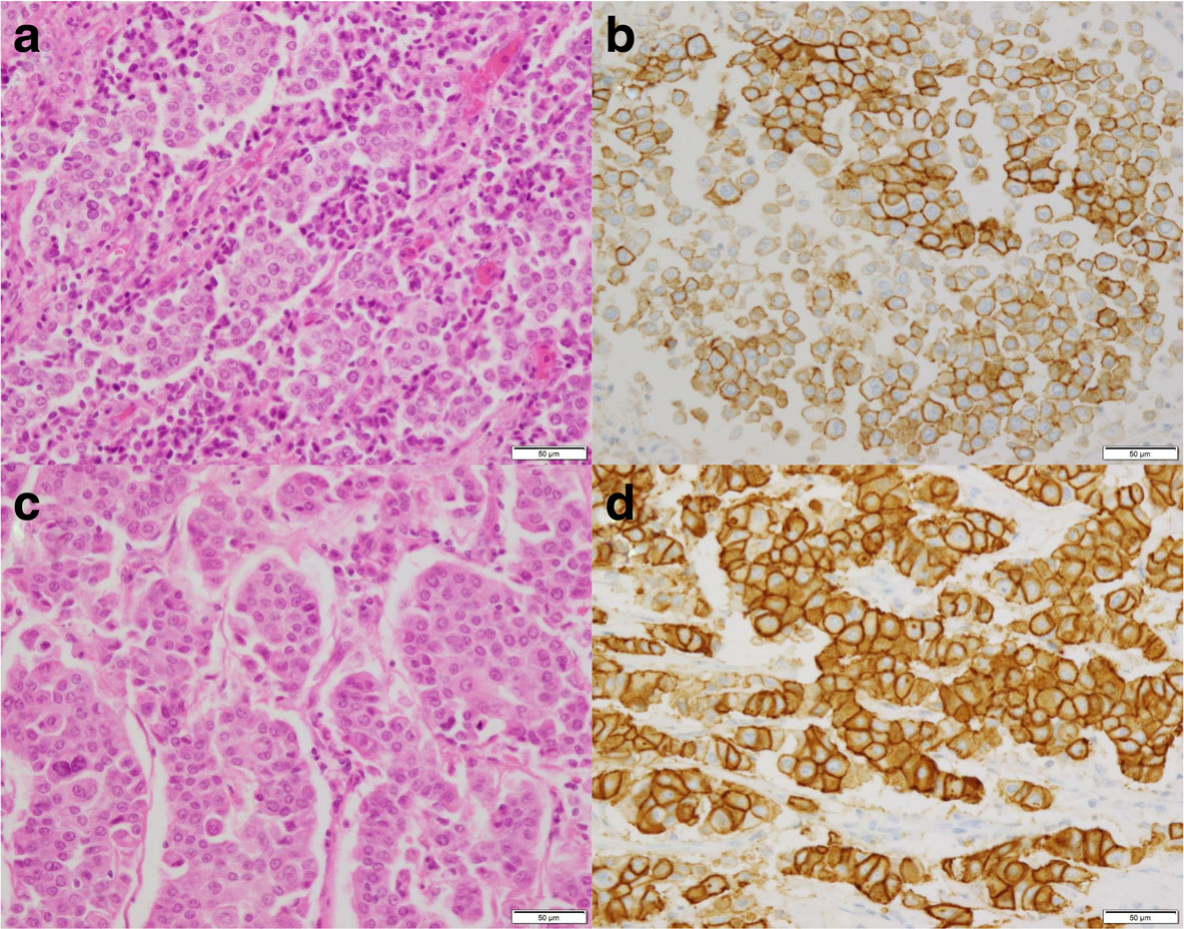
Histopathologic examination of the renal pelvic cancer and renal capsule cancer. **a** Hematoxylin and eosin (H&E)-stained section of the pelvic cancer. **b** H&E-stained section of the renal capsule cancer. **c** HER2-stained section of the pelvic cancer showing positive staining. **d** HER2-stained section of the renal capsule cancer showing positive staining

Immunohistochemically, the cancer cells of the renal pelvis were similar to those of the renal capsule (Fig. 2), whereas the cancer cells of the ureter were similar to those of the breast cancer (Table 1). We concluded that the renal capsule cancer was derived from the renal pelvic cancer, and the ureter cancer was derived from the breast cancer.

**Table 1:** Immunohistochemical examination

	HER2	ER	CK7
Renal pelvic cancer	+	–	+
Renal capsule cancer	+	–	+
Ureter cancer	–	+	±
Breast cancer	–	+	±

*HER* human epidermal growth factor receptor 2, *ER* estrogen receptor, *CK* cytokeratin

We thus diagnosed right pelvic cancer (urothelial carcinoma, G2 > G3, pT3) and renal capsule metastasis. We explained the necessity of adjuvant chemotherapy for the metastatic renal pelvic cancer to the patient, but she rejected chemotherapy and continued only with endocrine therapy. She then developed bilateral pleural effusions, mediastinal lymph node metastasis, para-aortic lymph node metastasis, and liver metastasis. She underwent a thoracentesis in October 2015, and the pleural effusion cytology was class V (Papanicolaou classification), compatible with breast cancer. Endocrine therapy was changed to letrozole 2.5 mg, but she died of breast cancer progression in January 2016.

## Discussion

Metastatic renal cancer is relatively common, and autopsy studies indicate that 12% of patients who die of cancer have renal metastasis [2]. Mostly, the metastases are to the renal parenchyma, and few are to the renal capsule. Most renal metastases develop via a hematogenous route and are derived from lung, breast, and gastrointestinal carcinoma, malignant melanoma, and hematologic malignant cancer [1–4]. Renal metastasis is generally accompanied by systemic metastasis, and renal metastatic lesions are often small and multifocal [6].

The renal capsule is a fibrous membrane that surrounds the renal parenchyma and can be separated from the renal parenchyma [7]. Primary renal capsule cancer is relatively rare. It originates from renal capsule structures and is derived from mesenchymal components, and almost all renal capsule cancer is sarcoma such as leiomyosarcoma [8]. Because high-grade renal sarcoma often grows rapidly, it is difficult to distinguish from sarcomatoid RCC in the clinical presentation and radiographic findings. The clinical presentation of renal sarcoma is similar to that of rapidly growing RCC, i.e., a palpable mass, abdominal or flank pain, and hematuria. In patients with renal capsule cancer, renal sarcoma should also be considered [9]. Wide local excision with negative margins is desirable in the case of localized renal sarcoma because the most important prognostic factors for renal sarcoma are margin status and tumor grade [8]. Contrastingly, metastatic renal capsule cancer is very rare and has never been reported in detail.

The *HER2* gene is an oncogene that has a similar structure to the epidermal growth factor receptor gene. Overexpression of HER2 protein occurs in 20% of patients with breast cancer and 13.5% of patients with upper urinary tract urothelial carcinoma [10, 11]. In the present patient, we concluded that the renal capsule metastasis was derived from the HER2-positive renal pelvic cancer, and the ureteral metastasis was derived from the HER2-negative breast cancer.

Ureter metastasis derived from breast cancer was reported in 7.8% of patients with disseminated breast cancer in an autopsy series [12] and can cause ureteral obstruction and renal insufficiency. It occurs in long-standing hormonal-dependent breast cancer with bone metastasis. The prognosis after diagnosis is relatively poor [13]. However, 8 of 15 (53%) patients with ureter metastasis derived from breast cancer did not show any clinical findings of ureteral obstruction [14]. The present patient did not have right hydronephrosis at surgery.

During the preoperative assessment of this patient, we diagnosed her renal capsule cancer as renal cell cancer because it was solitary and showed a contrast effect in the early phase of CT. Based on the pathological findings, we concluded that the renal capsule cancer was derived from the renal pelvic cancer.

## Conclusion

Renal capsule metastasis derived from renal pelvic cancer is very rare. When diagnosing renal capsule cancer, we believe that renal capsule metastasis should also be taken into consideration. Clinical and radiological differential diagnosis of renal capsule metastasis from renal cell cancer and primary renal capsule cancer is difficult. For this reason, assessment of the histopathological findings of the surgical specimens seems to be the only realistic approach to achieving the correct diagnosis.

## Abbreviation

CT: Computed tomography
